# Supplementary material for: Synergistic Anti-Inflammatory Effects of Lipophilic Grape Seed Proanthocyanidin and Camellia Oil Combination in LPS-Stimulated RAW264.7 Cells
Source: Antioxidants (Basel). 2022 Jan 31;11(2):289. doi: 10.3390/antiox11020289 (PMC8868477; doi:10.3390/antiox11020289)
Supplement: Supplementary file 1 [file antioxidants-11-00289-s001.zip › antioxidants-1547971-supplementary.pdf]

antioxidants

MDPI

Article

Synergistic anti-inflammatory effects of lipophilic grape seed proanthocyanidin and camellia oil combination in LPS-stimulated RAW264.7 cells

Linli Zhang [1], Juan Chen [2], Ruihong Liang [1], Chengmei Liu [1], Mingshun Chen [1,*] and Jun Chen [1]

[1] State Key Laboratory of Food Science and Technology, Nanchang University, Nanchang 330047, China; 1453049838@qq.com (L.Z.); liangruihong@ncu.edu.cn (R.L.); liuchengmei@ncu.edu.cn (C.L.); chenjun@ncu.edu.cn (J.C.)
[2] Moutai Institute, Renhuai 564501, China; kilooy@163.com
* Correspondence: chenshun1221@163.com; Tel.: +86 0791 88305871

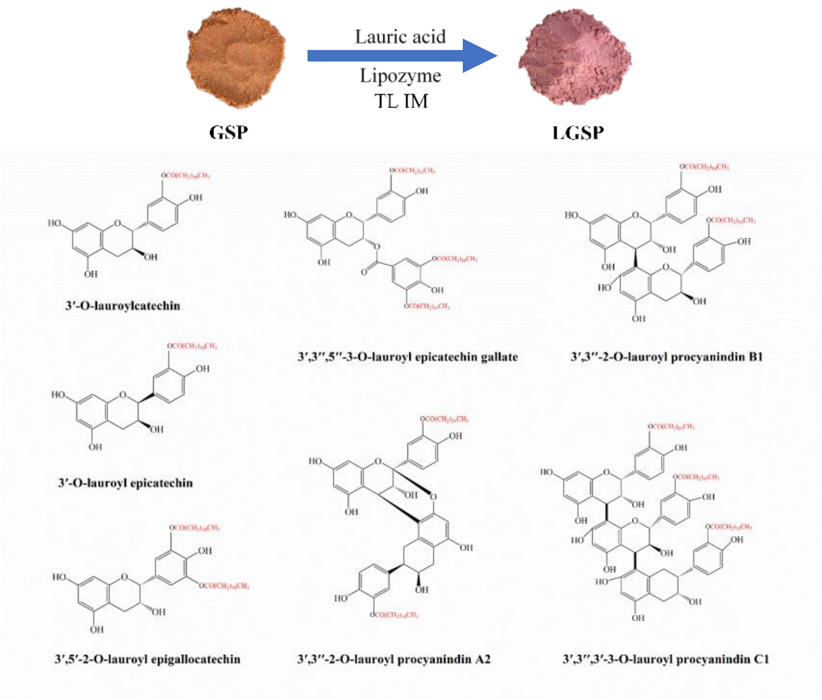

Figure S1. Effect of LGSP (A), CO (B) and their combination (C) on viability of RAW264.7 cells. The cells were treated with the indicated concentration of LGSP and/or CO for 24 h, and the cell viability was determined by MTT assay. The values represent the means ± SD from three independent experiments. Different letters represent significant differences ($p < 0.05$).
